# Mecamylamine inhibits seizure-like activity in CA1-CA3 hippocampus through antagonism to nicotinic receptors

**DOI:** 10.1371/journal.pone.0240074

**Published:** 2021-03-12

**Authors:** Olha Zapukhliak, Olga Netsyk, Artur Romanov, Oleksandr Maximyuk, Murat Oz, Gregory L. Holmes, Oleg Krishtal, Dmytro Isaev

**Affiliations:** 1 Department of Cellular Membranology, Bogomoletz Institute of Physiology, Kiev, Ukraine; 2 Faculty of Pharmacy, Department of Pharmacology and Therapeutics, Kuwait University, Safat, Kuwait; 3 Neurological Sciences, Larner College of Medicine, University of Vermont, Burlington, Vermont, United States of America; Georgia State University, UNITED STATES

## Abstract

Cholinergic modulation of hippocampal network function is implicated in multiple behavioral and cognitive states. Activation of nicotinic and muscarinic acetylcholine receptors affects neuronal excitability, synaptic transmission and rhythmic oscillations in the hippocampus. In this work, we studied the ability of the cholinergic system to sustain hippocampal epileptiform activity independently from glutamate and GABA transmission. Simultaneous CA3 and CA1 field potential recordings were obtained during the perfusion of hippocampal slices with the aCSF containing AMPA, NMDA and GABA receptor antagonists. Under these conditions, spontaneous epileptiform discharges synchronous between CA3 and CA1 were recorded. Epileptiform discharges were blocked by addition of the calcium-channel blocker Cd^2+^ and disappeared in CA1 after a surgical cut between CA3 and CA1. Cholinergic antagonist mecamylamine abolished CA3-CA1 synchronous epileptiform discharges, while antagonists of α7 and α4β2 nAChRs, MLA and DhβE, had no effect. Our results suggest that activation of nicotinic acetylcholine receptors can sustain CA3-CA1 synchronous epileptiform activity independently from AMPA, NMDA and GABA transmission. In addition, mecamylamine, but not α7 and α4β2 nAChRs antagonists, reduced bicuculline-induced seizure-like activity. The ability of mecamylamine to decrease hippocampal network synchronization might be associated with its therapeutic effects in a wide variety of CNS disorders including addiction, depression and anxiety.

## Introduction

Acetylcholine (ACh) exerts a wide range of neuromodulatory effects in numerous physiological and pathological states [1]. The action of ACh is mediated by two types of receptors: muscarinic (mAChRs) and nicotinic (nAChRs), named after their respective agonists muscarine and nicotine. While the muscarinic type G-protein coupled receptors (GPCRs) mediate a slow metabolic response via second-messenger cascades, the nicotinic type are ligand-gated ion channels that mediate fast cholinergic synaptic transmission [2,3]. In the hippocampus several subtypes of nicotinic (α7, α4β2 α3β4) and muscarinic (M1-M4) acetylcholine receptors are widely expressed in pyramidal cells and interneurons at pre- and postsynaptic sites [4,5]. Activation of AChRs has an important role in hippocampal hypersynchronization and pacing of neuronal activity [6,7]. The cholinergic agonist carbachol induces rhythmic oscillations that resemble patterns of epileptiform activity in vitro [8–10]. Epileptiform activity in brain slices represents electrographic correlates of epileptic seizures in vivo. The location, frequency, morphology and clinical significance of epileptic discharges varies widely, sometime complicating EEG interpretation in clinical practice [11]. The cholinergic agonist pilocarpine induces status epilepticus in vivo and recently it was shown that administration of pilocarpine causes a 6-fold increase of hippocampal ACh release paralleling the development of tonic seizures [12–14]. High doses of nicotine also induce seizures in animals, and mutations in genes coding for nAChR subunit are associated with seizures in humans [15–17]. Despite these multiple links to epilepsy, the exact function of cholinergic receptors in patterning of hippocampal synchronization remains unclear.

Synchronization of hippocampal fields is primarily mediated by glutamatergic and GABAergic synaptic transmission. We hypothesized that the widely distributed in hippocampus acetylcholine system is able to sustain synchronous synaptic synchronization of CA3 and CA1 zones. Due to the dominant role of glutamatergic and GABAergic systems in hippocampal synchronization, the impact of endogenous ACh is easily obscured during field potential recordings. The aim of this study was to investigate the ability of cholinergic neuromodulation to sustain hippocampal field synchronization in the absence of GABAergic and glutamatergic transmission. Simultaneous field potential recordings were obtained in CA3 and CA1 during perfusion of hippocampal slices with aCSF containing AMPA, NMDA and GABA receptor antagonists. Cholinergic antagonists were added to the perfusion solution to study the effect of AChRs activation during hippocampal field synchronization. We also compared the effects of the nicotinic antagonist mecamylamine (MEC) and selective α7 and α4β2 nAChRs antagonists on induced hippocampal seizure-like activity (SLA).

## Materials and methods

### Animals

All experimental procedures were performed on Wistar rats according to the guidelines provided by the National Institutes of Health for the humane treatment of animals and approved by the Animal Care Committee of Bogomoletz Institute of Physiology of National Academy of Science of Ukraine. Postnatal day 10–14 rats were deeply anesthetized using sevoflurane and decapitated. Transverse brain slices were prepared according to previously described techniques [18]. Briefly, brains were removed and placed in the ice-cold aCSF of the following composition (in mM): 126 NaCl, 3.5 KCl, 2 CaCl_2_, 1.3 MgCl_2_, 1.25 NaH_2_PO_4_, 24 NaHCO_3_, 11 D-glucose, pH = 7.35–7.4, the pH of all aCSF solutions was adjusted with carbogen gas. Hippocampal slices were cut at 500 μm using a Vibroslice NVSL (World Precision Instruments, Sarasota, FL). Slices equilibrated at room temperature and constantly carbogenated aCSF for at least two hours before the experiment. Transverse hippocampal slices with a clearly visible pyramidal cell layer in CA3-CA1 zones were selected for electrophysiological experiments. Under microscopic control, the recording electrodes were positioned in the pyramidal cell layer in CA3 and CA1 zones.

### Induction of epileptiform activity

Synchronous epileptiform discharges were induced by perfusion of hippocampal slices with a low-Mg^2+^ aCSF containing AMPA, NMDA and GABA receptor antagonists, referred to as “synaptic blockers aCSF”. Synaptic blockers aCSF had the following composition (in mM): 100 NaCl, 5 KCl, 1 CaCl_2_, 1.25 NaH_2_PO_4_, 24 NaHCO_3_, 11 D-glucose; and 6,7-dinitroquinoxaline-2,3-dione (DNQX 10μM); S,10R)-(+)-5-methyl-10,11-dihydro-5H-dibenzo[a,d]cyclohepten-5,10-imine maleate (MK-801 2 μM); [R-(R*,S*)]-6-(5,6,7,8-tetrahydro-6-methyl-1,3-dioxolo[4,5-g]isoquinolin-5-yl)furo[3,4-e]-1,3-benzodioxol-8(6H)one (bicuculline 10μM).

Nonsynaptic SLA was induced by perfusion of the hippocampal slices with low-Ca^2+^ aCSF of the following composition (in mM): 115 NaCl, 5 KCl, 1 MgCl_2_, 1.25 NaH2PO_4_, 24 NaHCO_3_, 11 D-glucose.

Bicuculline (10 μM) and 4-aminopyridine (4-AP, 100μM) were used to induce SLA in the following aCSF (in mM): 125 NaCl, 5 KCl, 1 CaCl_2_, 1.3 MgCl_2_, 1.25 NaH_2_PO_4_, 11 D-glucose, 24 NaHCO_3_. Chemicals were purchased from Sigma (St. Louis, MO), DNQX, DhβE, d-tubocurarine were obtained from Tocris (Ellisville, MO).

### Extracellular and patch clamp recordings

For extracellular recordings slices were transferred to a submerged recording chamber and perfused with oxygenated aCSF (22–25°C) at a rate of 2–3 ml*min^-1^. Temperature control was performed with the Dual Temperature Controller (TC-144, Warner Instruments). Simultaneous recordings of field potentials were obtained from the CA3 and CA1 pyramidal cell layer with extracellular glass microelectrodes (2–3 MΩ) filled with aCSF. Signals were low-pass filtered (0.5 kHz), amplified using a 2-channel differential amplifier M1800 (A-M Systems, Carlsborg, WA), digitized at 10 kHz using an analog-to-digital converter (NI PCI-6221; National Instruments, Austin, TX), extracellular recording were made using WinWCP program (Strathclyde Electrophysiology Software, University of Strathclyde, Glasgow, UK).

Patch clamp recordings were performed using an RK-400 amplifier (BioLogic, France) digitally sampled at 10 kHz and filtered at 3 kHZ simultaneously with extracellular recording to investigate the coincidence of postsynaptic currents and epileptiform discharges. CA1 pyramidal cells were visually identified with an infrared-differential interference contrast (IR-DIC) microscope (Olympus BX50WI) and captured with a CoolSNAP ES2 (CCD ICX285) video camera. Spontaneous postsynaptic currents were recorded from CA1 pyramidal cells using a patch clamp technique in a whole-cell configuration. Records were made using the WinWCP program (Strathclyde Electrophysiology Software, University of Strathclyde, Glasgow, UK). Patch electrodes were fabricated from borosilicate glass capillaries of 1.5 mm outer diameter (Sutter Instruments, USA) using a programmable puller (P-97; Sutter Instruments, USA). The recording pipettes were filled with (in mM): 100 Cs-gluconate, 17.5 CsCl, 8 NaCl, 10 HEPES, 10 EGTA, 2 MgATP (pH 7.3). When filled with intracellular solution, recording pipettes typically had resistances of 5–7 MΩ.

### Data analysis

Data were analyzed with Clampfit (Axon Instruments) and Origin 8.0 (OriginLab, Northampton, MA). Cross-correlation analysis was used to determine the level of synchronization between CA3 and CA1 field potential recordings. The sampling data of recordings were filtered by the low pass digital Gaussian filter with a cut-off frequency of 50 Hz. Cross-correlation function was then calculated for paired signal samples of simultaneous CA3-CA1 field potential recordings (about 600 s duration and no less than 300 s). Obtained cross-correlation functions were then smoothed using the Lowess smoother (span = 0.01). Next, the first maximum of each cross-correlation function was measured to estimate the level of synchronization of CA3-CA1 field potential. Results of CA3-CA1 synchronization was reported as a mean max cross-correlation value ± standard deviation. For data comparison, the *t*-test was used for data with a normal distribution (which was determined with the Shapiro-Wilk test for normality), otherwise the Kolmogorov-Smirnov test was used for comparison between unpaired samples and the Wilcoxon signed-rank test was used for statistical analysis of paired samples. Results obtained from simultaneous CA3-CA1 dual field potential recordings from 127 slices (69 rats) are reported with the *n* indicating the number of slices with simultaneous CA3-CA1 field potential recordings, unless otherwise stated. Summary data are presented as mean ± SD and p < 0.05 was considered statistically significant.

## Results

### Spontaneous epileptiform discharges synchronized between CA3 and CA1 in aCSF with AMPA, NMDA and GABA antagonists

**Fig 1:**
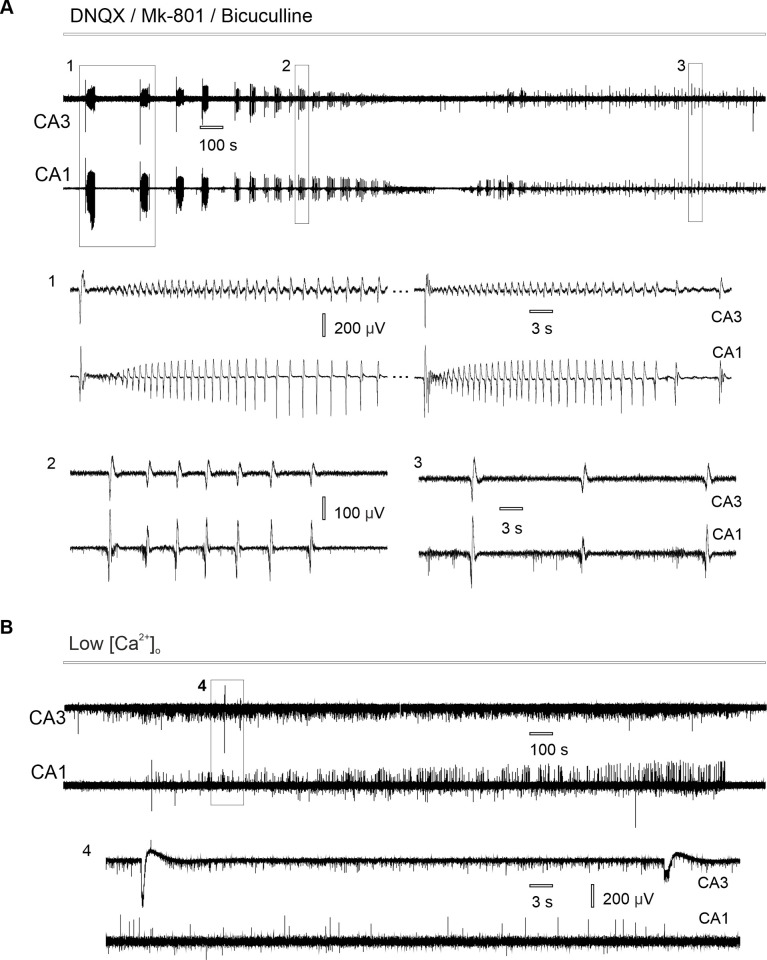
Spontaneous hippocampal epileptiform discharges in CA3 and CA1 under nonsynaptic conditions. (A) Simultaneous recording of synchronous epileptiform activity induced in synaptic blockers aCSF: (1) bursts of discharges, (2) single epileptiform discharges, (3) epileptiform discharges appear simultaneously in CA3 and CA1, while population spikes only in CA1. (B) Nonsynaptic population spikes appear in low-Ca^2+^ aCSF: (4) epileptiform activity is not synchronized between CA3-CA1 hippocampal zones.

Perfusion of hippocampal slices with aCSF containing AMPA, NMDA and GABA antagonists (DNQX 10μM, MK-801 2μM, bicuculline 10μM) induced spontaneous epileptiform activity that developed simultaneously in CA3 and CA1 zones (n = 25 slices from 14 rats with simultaneous CA3-CA1 field potential recording). The recorded activity represented spontaneous *epileptiform discharges* (Fig 1) that had a mean duration of 1.07 ± 0.34 sec (n = 100 events from 20 slices from 12 rats) and appeared with a rate of 2.36 ± 1.11 events per minute (n = 20 slices from 12 rats,). In 48% of slices (12/25) epileptiform events also appeared in bursts–trains of discharges that had a mean duration of 35.61 ± 12.67 sec (n = 45 bursts from 12 slices from 9 rats) and mean frequency 0.52 ± 0.28 Hz (n = 45 bursts from 12 slices from 9 rats, Fig 1B). A burst of discharges usually started with a large amplitude field event followed by a train of epileptiform discharges (Fig 1A–1).

While hippocampal slices are known to produce epileptiform bursting under nonsynaptic conditions such as low-Ca^2+^ milieu [19–21], we hypothesized that different, synaptic mechanisms account for epileptiform discharges synchronization in synaptic blockers aCSF, unlike nonsynaptic mechanisms of low-Ca^2+^ SLA (Fig 1B).

To evaluate the level of CA3-CA1 field potential synchronization in synaptic blockers aCSF we used cross-correlation for the simultaneous CA3-CA1 field potential recordings. During perfusion with the synaptic blockers aCSF field potentials were synchronized between CA3 and CA1 (cross correlation 0.47 ± 0.17/n = 25 slices, 14 rats). However, in low-Ca^2+^ aCSF the level of CA3-CA1 synchronization was significantly lower (cross-correlation 0.05 ± 0.04, n = 12 slices from 8 rats, Kolmogorov-Smirnov test p ˂ 0.001, Fig 1B).

**Fig 2:**
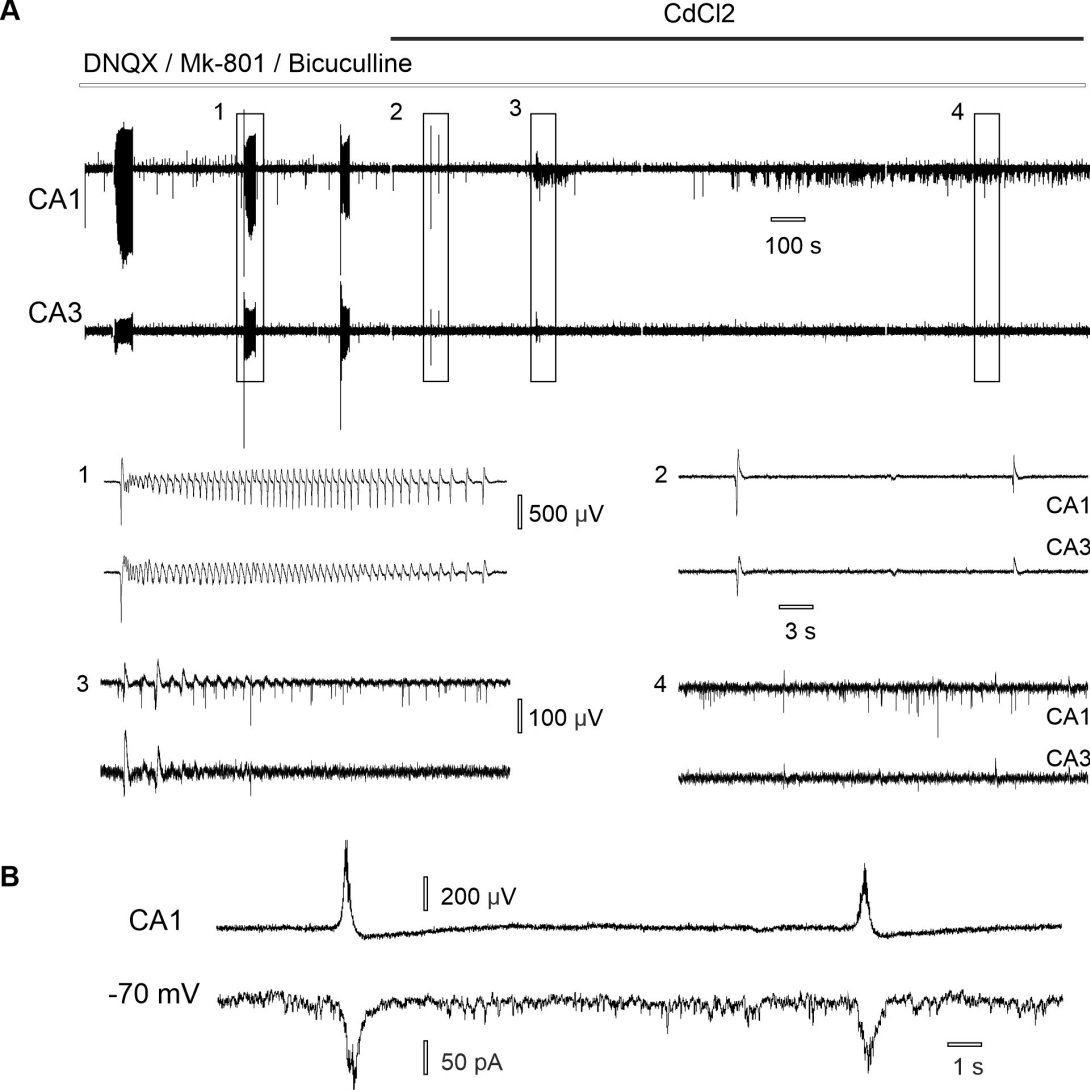
CA3-CA1 synchronization of epileptiform discharges induced in aCSF with AMPA, NMDA, GABA antagonists depends on synaptic connections. (A) Blockade of the synchronous epileptiform discharges following 15 μM CdCl_2_ application: (1) burst of epileptiform discharges and (2) single epileptiform discharges become less synchronized (3) following 15 μM CdCl_2_ application and nonsynaptic population spikes (4) appear in CA1. (B) Simultaneous extracellular (upper trace) and intracellular (bottom trace) recording in CA1 during perfusion with synaptic blockers aCSF reveals synaptic currents appear during epileptiform discharges.

**Fig 3:**
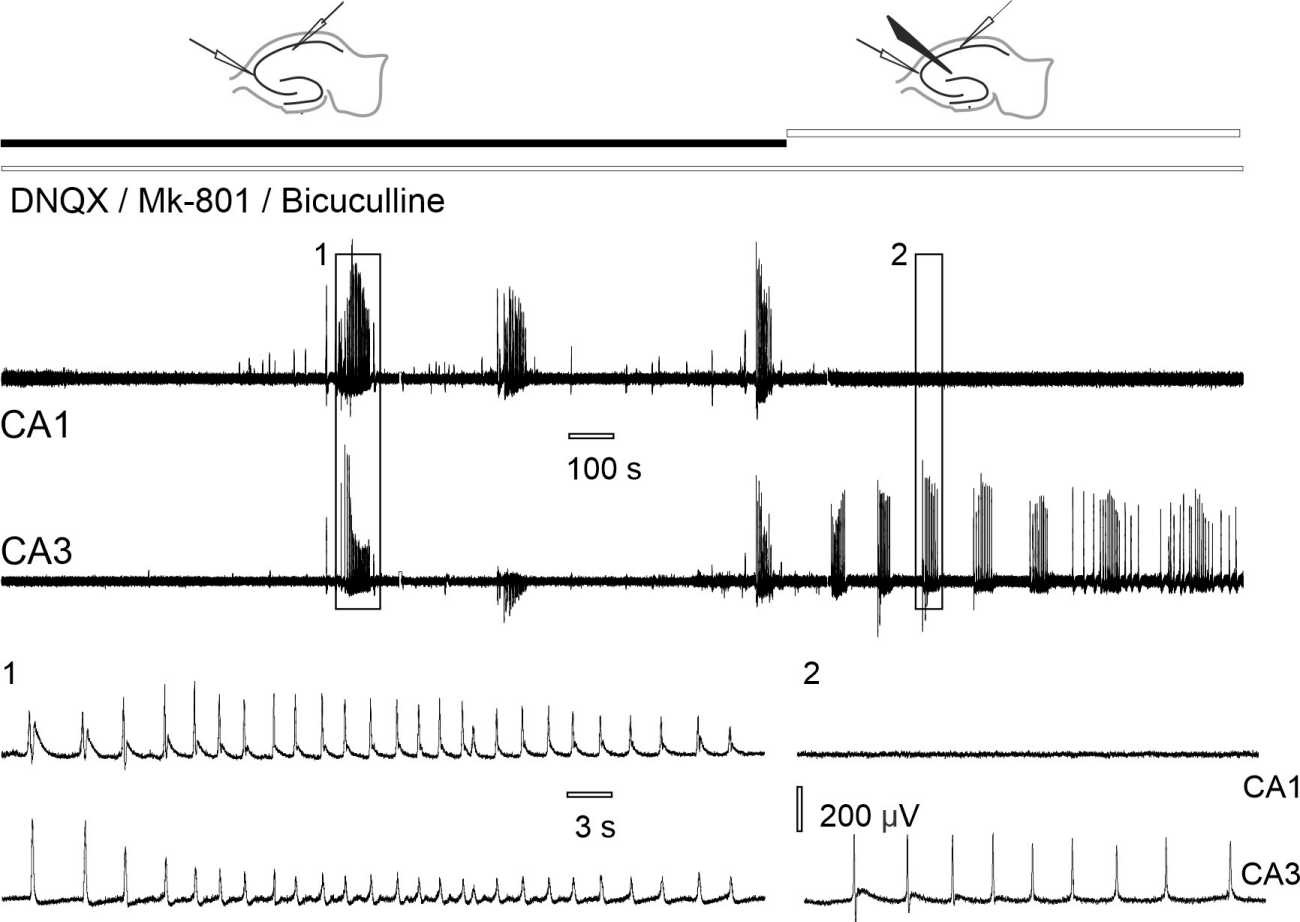
Effect of mechanical separation of CA3 and CA1 hippocampal zones on epileptiform discharges in synaptic blockers aCSF. (1) Burst of discharges synchronized between CA3-CA1. (2) Epileptiform discharges disappear in CA1, but not in CA3 after the separation was made between two recording sites.

**Fig 4:**
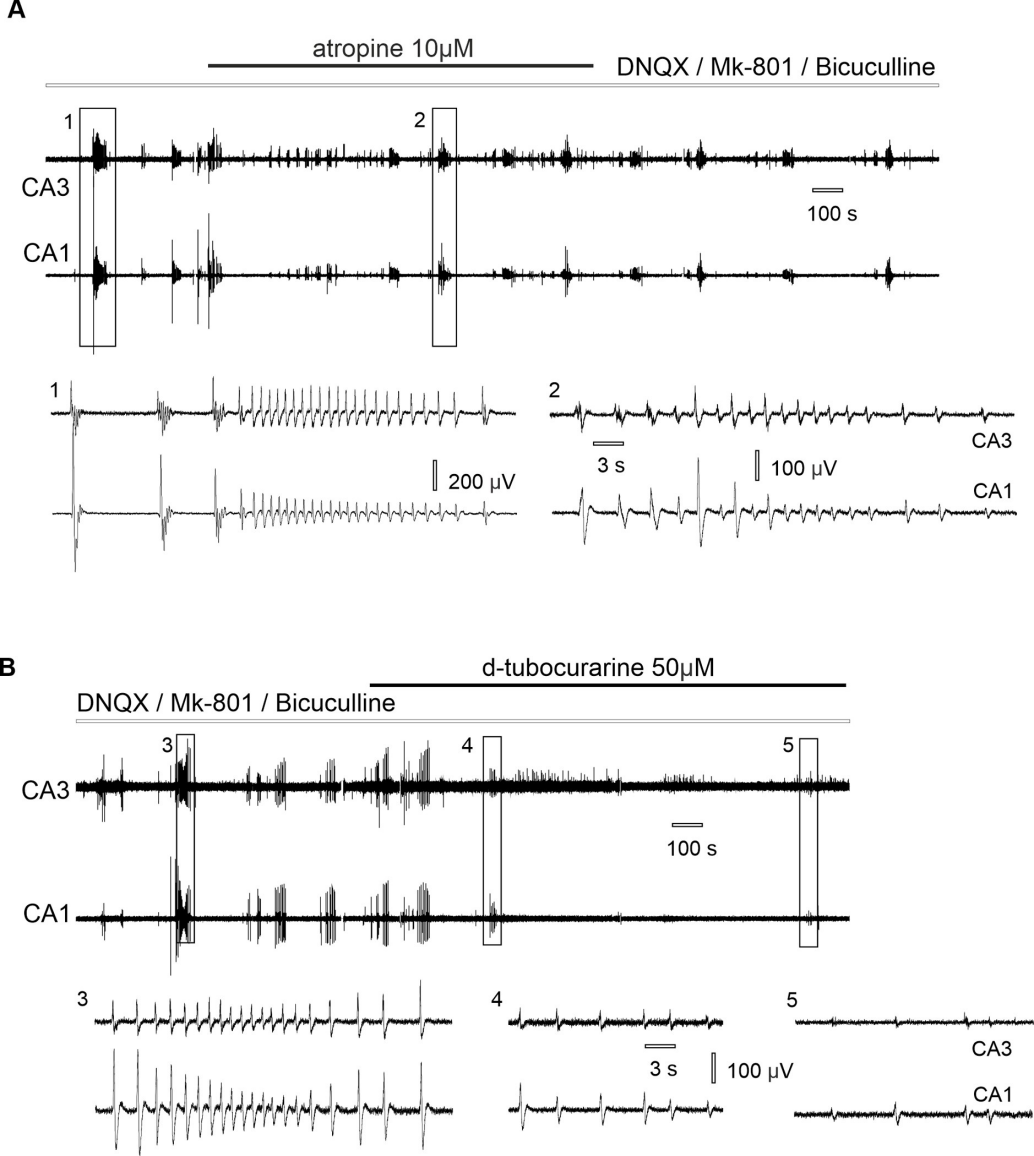
Effect of cholinergic antagonists on CA3-CA1 synchronous epileptiform discharges induced in synaptic blockers aCSF. (A) Application of atropine does not block CA3-CA1 synchronous discharges: (1) burst of discharges in synaptic blockers aCSF, (2) synchronous epileptiform discharges appear during atropine application. (B) Application of d-tubocurarine blocks CA3-CA1 synchronous epileptiform discharges: (3) burst of discharges in synaptic blockers aCSF, (4) reduction of the discharges after tubocurarine application, (5) epileptiform discharges decrease under tubocurarine application.

Application of CdCl_2_ abolished synchronous epileptiform discharges induced in synaptic blockers aCSF (Fig 2A). Following 10 min of stable CA3-CA1 synchronous epileptiform bursting (cross-correlation 0.44 ± 0.14), 15 μM CdCl_2_ was added to the perfusion aCSF, which resulted in complete blockade of synchronous discharges (cross-correlation 0.06 ± 0.02, n = 22 slices from 11 rats, Shapiro-Wilk test, paired *t*-test p ˂ 0.001). As epileptiform discharges are irregular and have low frequency we cannot be sure of exact delay time of the Cd^2+^ effect. In 10/22 (45.5%) epileptiform discharges never appeared after the start of the perfusion of CdCl_2_-aCSF. In 12/22 (54.5%) slices discharges were blocked following a mean delay time of 4.09 ± 2.60 min. Prolonged perfusion with 15 μM CdCl_2_-synaptic blockers aCSF resulted in the development of nonsynaptic population spikes (n = 10 slices, 8 rats, Fig 2A–4), which were similar to number of population spikes in low-Ca^2+^ aCSF (Fig 1B).

To further prove synaptic origin of the recorded epileptiform discharges neurons were voltage-clamped at a holding potential of -70 mV. Simultaneous patch-clamp and field potential recording during perfusion with synaptic blockers aCSF revealed the coincidence of synaptic currents (mean amplitude 51.60 ± 22.71 pA, n = 81 events/7 neurons, 7 slices from 4 rats) with the epileptiform discharges (Fig 2B). Application of 15 μM CdCl_2_ on hippocampal slices blocked epileptiform discharges and corresponding synaptic currents (n = 3 neurons, 3 slices/2 rats).

Mechanical separation of CA3 and CA1 hippocampal fields resulted in complete abolishment of epileptiform discharges in CA1 but not in CA3 (cross-correlation 0.51 ± 0.21, after the surgical cut– 0.13 ± 0.07, n = 10 slices from 7 rats, Wilcoxon signed ranks test, p = 0.002, Fig 3).

#### nAChRs contribute to CA3-CA1 synchronization of epileptiform discharges in aCSF with AMPA, NMDA, GABA antagonists

Application of the muscarinic antagonist atropine (10 μM) did not abolish synchronous epileptiform discharges (Fig 4A and Table 1). Synchronous discharges were blocked following application of the nicotinic antagonist d-tubocurarine (50 μM, Fig 4B) suggesting the role of nAChRs in observed epileptiform activity.

**Table 1:** Effect of cholinergic antagonists on CA3-CA1 synchronization of epileptiform discharges.

Cholinergic antagonist	Cross-correlation control	Cross-correlation application	Statistical significance (Wilcoxon signed ranks test)		Number of slices
atropine, 10 μM	0.56 ± 0.15	0.38 ± 0.19	-	p = 0.059	n = 6
d-tubocurarine, 50 μM	0.55 ± 0.25	0.13 ± 0.05	*	p = 0.036	n = 6
MLA, 100 nM	0.56 ± 0.08	0.31 ± 0.18	-	p = 0.059	n = 5
DhβE, 10 μM	0.48 ± 0.22	0.43 ± 0.33	-	p = 0.850	n = 4
MEC, 50 μM	0.45 ± 0.18	0.10 ± 0.09	**	p = 0.002	n = 12

**Fig 5:**
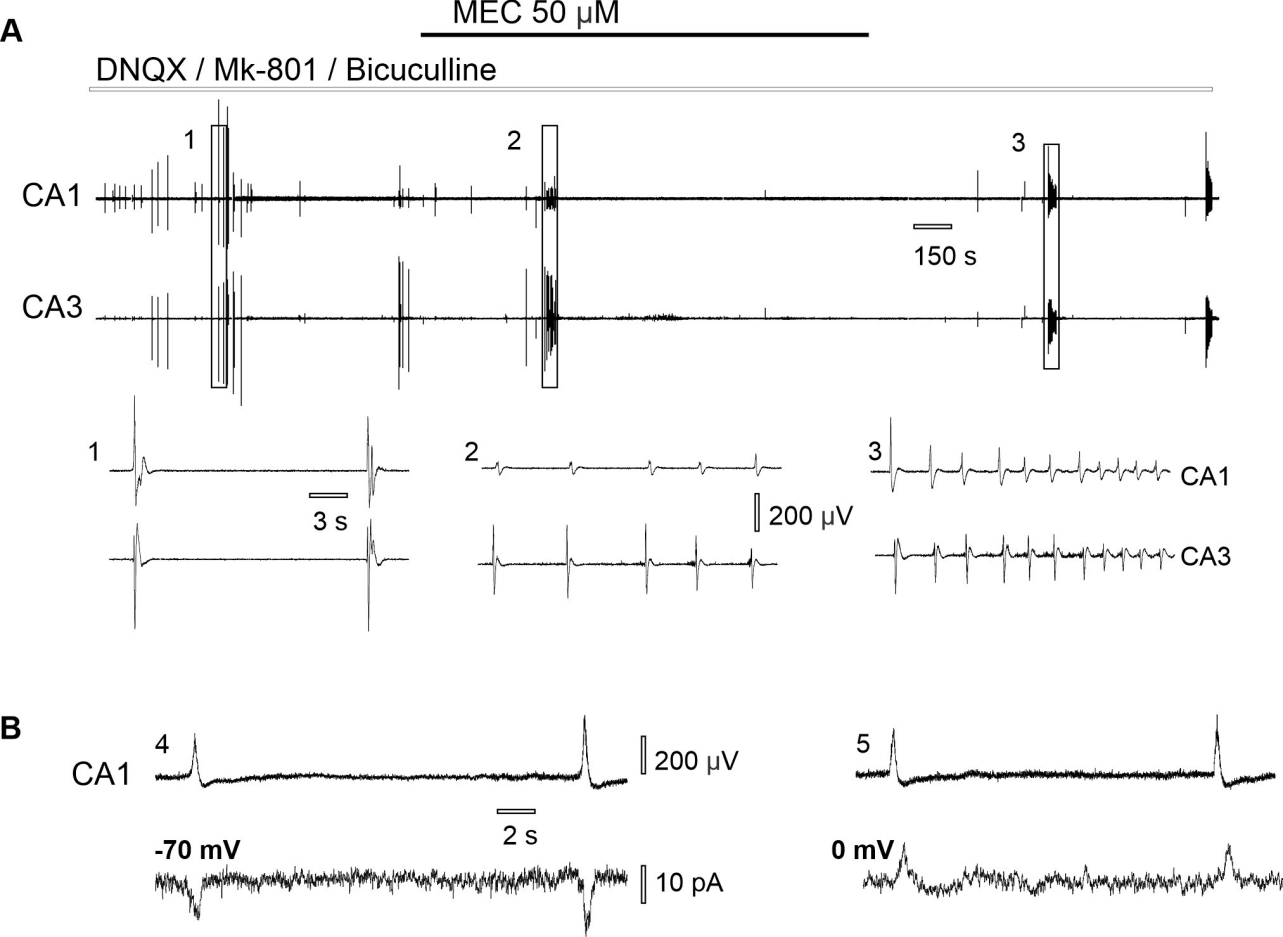
Nicotinic receptors contribute to synchronous discharges in synaptic blockers aCSF. (A) Effect of nonselective nicotinic antagonist mecamylamine on CA3-CA1 synchronous epileptiform discharges induced in synaptic blockers aCSF: (1) single epileptiform discharges; (2) decrease in epileptiform discharges following 50 μM MEC application; (3) epileptiform discharges after mecamylamine wash out. (B) Simultaneous extracellular (up) and patch clamp recording (bottom) in synaptic blockers aCSF: (4) neuron voltage clamped at -70 mV, (5) neuron voltage clamped at zero mV.

Application of the nonselective nicotinic antagonist mecamylamine (50 μM) resulted in complete abolishment of epileptiform discharges after some latency and significant reduction of CA3-CA1 synchronization (p = 0.002, Table 1 and Fig 5A).

Considering the reversal potential of the nicotinic receptor’s response (E_ACh_ = 0 mV), we performed simultaneous extracellular recording of epileptiform discharges and voltage clamp neurons at 0 mV (n = 7 cells, 7 slices/4 rats, Fig 5B). During the recording at 0 mV of 76 epileptiform discharges we detected only 14 simultaneous synaptic currents with an averaged amplitude of -1.92 ± 14.89 pA, while at the holding potential of -70 mV synaptic currents appeared in response to all recorded epileptiform discharges (86 out of 86 and average amplitude of synaptic currents at the holding membrane potential -70mV was 51.60 ± 22.71 pA, Fig 5B). Application of 50 μM MEC also abolished synaptic currents that coincided with epileptiform discharges (n = 4 neurons, 4 slices from 2 rats).

**Fig 6:**
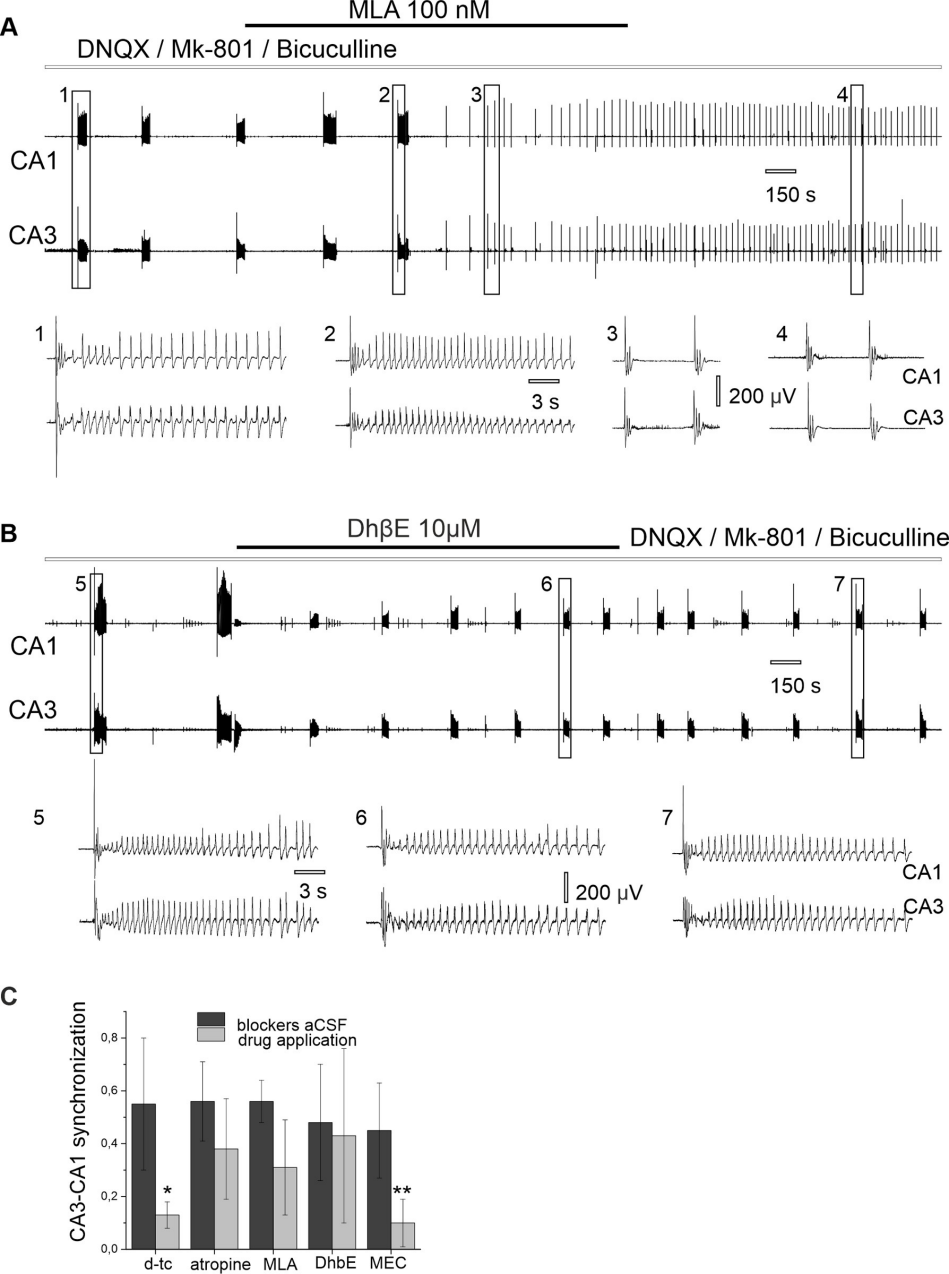
Effect of selective nicotinic antagonists on spontaneous epileptiform discharges in synaptic blockers aCSF. (A) Application of α7 nAChRs antagonist MLA (100 nM) does not stop synchronous discharges: (1) burst of discharges in synaptic blockers aCSF; (2) burst of discharges during MLA application; (3) single field discharges in presence of MLA and (4) after wash out. (B) Synchronous burst of discharges appears in (1) synaptic blockers aCSF, (2) after DhβE application, and after perfusion was returned to synaptic blockers aCSF (3). (C) Summary data of the effect of cholinergic antagonists on cross-correlation between CA3 and CA1 field potential in synaptic blockers aCSF.

Application of α7 and α4β2 nAChRs antagonists, 100 nM MLA and 10 μM DhβE respectively had no substantial effect on CA3-CA1 synchronization (Fig 6A and 6B). Results are presented in Table 1.

### Effect of MEC on hippocampal SLA induced by bicuculline and 4-AP

**Fig 7:**
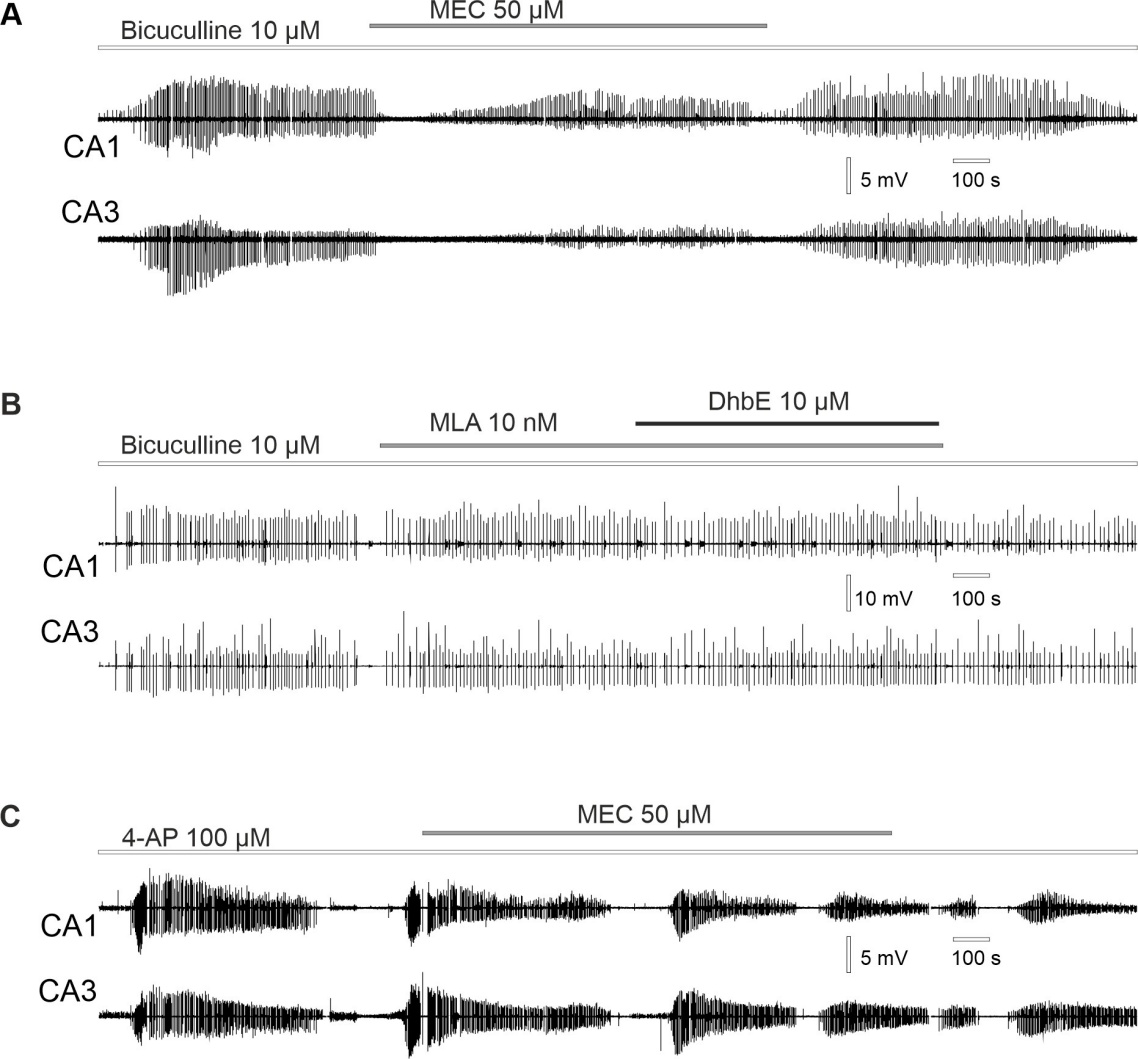
Effect of the cholinergic antagonists on hippocampal SLA activity. **(**A) Reduction of the bicuculline-evoked SLA following MEC application. (B) Application of α7 nAChRs antagonist MLA and α4β2 antagonist DhβE has no effect on bicuculline-evoked SLA. (C) Application of MEC has no effect on 4-AP induced SLA.

Next, we tested MEC on its potential antiseizure properties in two models of SLA: bicuculline and 4-AP. Application of MEC (50 μM) has no significant effect on field potential synchronization during bicuculline-induced SLA (cross-correlation 0.50 ± 0.2 vs 0.57 ± 0.15, n = 10 slices from 4 rats, Shapiro-Wilk test, paired *t*-test p = 0.11). However, application of MEC significantly reduced the amplitude of bicuculline-induced SLA in CA3: 2.04 ± 1.04 mV vs 1.61 ± 0.95 mV (n = 10 slices from 4 rats, Wilcoxon signed ranks test p = 0.008); in CA1: 3.45 ± 1.87 mV vs 2.19 ± 1.13 mV (n = 10 slices, Wilcoxon signed ranks test p = 0.009, Fig 7A). There was no significant effect on the frequency of bicuculline-induced SLA following MEC application (0.13 ± 0.07 Hz vs 0.15 ± 0.08 Hz, n = 10 slices, 4 rats, Shapiro-Wilk test, paired *t*-test p = 0.3).

As expected, application of the selective α7 nAChRs antagonist MLA (100nM) and selective antagonist for α4β2 nAChRs DhβE (10μM) also had no effect on field potential synchronization during bicuculline-induced SLA (cross-correlation 0.31 ± 0.19 vs 0.28 ± 0.06, n = 3 slices from 2 rats, Wilcoxon signed ranks test p = 1). Likewise, application of α7 and α4β2 antagonists had no effect neither on the amplitude of bicuculline-induced SLA: in CA3 6.88 ± 3.04 mV vs 5.7 ± 3.1 mV (n = 3 slices from 2 rats, Wilcoxon signed ranks test p = 0.18); in CA1 5.03 ± 0.66 mV vs 4.38 ± 0.46 mV (n = 3 slices, Wilcoxon signed ranks test p = 0.18); nor on the frequency: 0.06 ± 0.01 Hz vs 0.06 ± 0.03 Hz (n = 3 slices, Wilcoxon signed ranks test p = 1, Fig 7B).

Also, application of mecamylamine has no effect on field potential synchronization during 4-AP-induced SLA (cross-correlation: 0.44 ± 0.17 vs 0.43 ± 0.2, n = 5 slices from 3 rats, Wilcoxon signed ranks test p = 0.78). Additionally, application of MEC had no significant effect neither on amplitude of 4-AP SLA: in CA3 3.49 ± 2.09 mV vs 2.93 ± 1.58 mV (n = 5 slices, Wilcoxon signed ranks test p = 0.58); in CA1 1.47 ± 0.45 mV vs 1.34 ± 0.61 mV (n = 5 slices, Wilcoxon signed ranks test p = 0.78); nor on the frequency: 0.36 ± 0.06 Hz vs 0.33 ± 0.07 (n = 5 slices, Wilcoxon signed ranks test p = 0.58, Fig 7C).

## Discussion

While most of hippocampal synaptic interactions are mediated by glutamate and GABA receptors, neuromodulation through other synaptic systems, such as ACh, exerts powerful effects on network function [22]. Considering the extreme hippocampal propensity for synchronization, we hypothesized that role of endogenous ACh in the hippocampal field potential synchronization might be detected under conditions of increased neuronal excitability and in the absence of AMPA, NMDA and GABA transmission. Since net electrical activity is mostly inhibited under these conditions, we increased neuronal excitability by decreasing osmolarity, omitting Mg^2+^ and increasing K^+^ concentration in perfusion aCSF, which was used by other laboratories to record non-synaptic SLA as described in earlier studies [18,19]. Under these conditions of increased neuronal excitability, we observed synchronous epileptiform discharges between CA3 and CA1.

Hippocampal networks can sustain robust SLA under nonsynaptic conditions, such as in the zero-Ca^2+^ milieu [21,23]. Perfusion of hippocampal slices with glutamate and GABA antagonists was shown to induce nonsynaptic bursting like low-Ca^2+^ discharges [20]. However, synchronization of discharges between hippocampal areas has never been observed under nonsynaptic conditions. In the present study, we report CA3-CA1 synchronous epileptiform discharges in the presence of AMPA, NMDA and GABA antagonists. Addition of CdCl_2_ completely abolished synchronous epileptiform discharges, indicating their dependence on voltage-gated calcium channel activation. After mechanical separation of CA1 from CA3, discharges remained in CA3 but disappeared in CA1, suggesting CA3 as generating site [10]. Simultaneous patch-clamp and field potential recordings of postsynaptic activity revealed coincidence between postsynaptic currents and epileptiform discharges. Taken together these results suggest that observed epileptiform discharges have synaptic origin.

ACh exerts multiple effects on hippocampal functioning through a wide range of nAChRs and mAChRs [24–26]. In the present study, atropine had no substantial effect on synchronization of epileptiform discharges. However, perfusion with the nicotinic antagonists d-tubocurarine or MEC completely abolished CA3-CA1 synchronous epileptiform discharges. These results suggest that activation of nAChRs is able to sustain hippocampal CA3-CA1 synchronization independently of AMPA, NMDA and GABA conductivities.

Three main types of nAChRs are described on hippocampal neurons, namely α7, α4β2, and α3β4 [27,28]. In our experiments, antagonists of α7 and α4β2 nAChRs–MLA and DhβE respectively–had no substantial effect on CA3-CA1 field synchronization. Meanwhile, MEC, a nonselective and noncompetitive nAChRs antagonist, readily abolished CA3-CA1 synchronous epileptiform discharges and blocked postsynaptic currents recorded during these events. MEC was initially developed as an antihypertensive medication but has been studied recently for its therapeutic potential in several neuropathological conditions [29,30]. Beneficial effects of MEC has been reported for epilepsy, substance abuse, depression and anxiety [31–34] and recently it was shown, that MEC reduces levels of ACh and decreases seizures in pilocarpine-induced status epilepticus in rats [12]. Here, we report that MEC has a substantial effect on hippocampal network synchronization, and that this effect cannot be explained by activation of α7 or α4 nAChRs subtypes, implying a possible role for the α3β4 subtype. We further studied the effects of MEC on SLA induced by bicuculline and 4-AP. Application of MEC did not change SLA in 4-AP model, suggesting that nAChRs are not involved in this model of epilepsy. However, MEC caused a significant decrease in the amplitude of bicuculline-induced SLA. Previously it was demonstrated that MEC effectively blocks seizures in maximal electroshock seizure test in mice, while antagonist of α7 receptors MLA does not prevent seizures in this model, however, MEC had no anticonvulsive properties in kindled rats, in which nAChRs antagonists exerted less robust effects [35]. The authors suggested that nAChRs antagonism could be a potential therapeutic approach to treat generalized epileptic seizures but rather not complex partial seizures [35]. In our work, application of α7 and α4β2 antagonists MLA and DhβE had no effect on bicuculline-induced SLA, further suggesting that the MEC effect on bicuculline-induced bursting is mediated not through α7 or α4β2 nAChRs subtypes. Thus, our results support the ability of MEC to decrease hippocampal network synchronization, which could partially explain the therapeutic effects of MEC in a wide variety of CNS disorders.

## Supporting information

S1 File (XLSX)
